# COVID-19 vaccine coverage, safety, and perceptions among patients with diabetes mellitus in China: a cross-sectional study

**DOI:** 10.3389/fendo.2023.1172089

**Published:** 2023-06-01

**Authors:** Haolong Li, Fan Ping, Xiaomeng Li, Zhihong Wang, Jianzhong Xiao, Hongwei Jiang, Yaoming Xue, Jinxing Quan, Hebin Yao, Xianling Zheng, Yanming Chen, Yufeng Li, Xiaohua Yu, Lingling Xu, Xinxin Feng, Siyu Wang, Yongzhe Li, Xinhua Xiao

**Affiliations:** ^1^ Department of Clinical Laboratory, State Key Laboratory of Complex Severe and Rare Diseases, Peking Union Medical College Hospital, Chinese Academy of Medical Science and Peking Union Medical College, Beijing, China; ^2^ Department of Endocrinology, Peking Union Medical College Hospital, Beijing, China; ^3^ Department of Medical Research Center, Peking Union Medical College Hospital, Chinese Academy of Medical Science and Peking Union Medical College, Beijing, China; ^4^ Department of Endocrinology, the First Affiliated Hospital of Chongqing Medical University, Chongqing, China; ^5^ Department of Endocrinology, Beijing Tsinghua Changgung Hospital, School of Clinical Medicine, Tsinghua University, Beijing, China; ^6^ The First Affiliated Hospital, College of Clinical Medicine of Henan University of Science and Technology, Luoyang, China; ^7^ Department of Endocrinology and Metabolism, Nanfang Hospital, Southern Medical University, Guangzhou, China; ^8^ Department of Endocrinology, Gansu Provincial People’s Hospital, Lanzhou, Gansu, China; ^9^ Department of Endocrinology, Sixth Medical Center of PLA General Hospital, Beijing, China; ^10^ Department of Endocrinology, Handan Central Hospital, Handan, China; ^11^ Department of Endocrinology & Metabolism, Guangdong Provincial Key Laboratory of Diabetology, The Third Affiliated Hospital of Sun Yat-sen University, Guangzhou, Guangdong, China; ^12^ Department of Endocrinology, Capital Medical University, Beijing, China; ^13^ Department of Endocrinology, Liaoyang Central Hospital, Liaoyang, Liaoning, China; ^14^ Department of Endocrinology, ShenZhen Hospital, Southern Medical University, Shenzhen, Guangdong, China

**Keywords:** diabetes mellitus, COVID-19, vaccine, vaccine uptake, China

## Abstract

**Aims:**

Diabetes mellitus (DM), one of the most common chronic diseases in China, is a risk factor for SARS-COV-2 infection and poor prognosis of COVID-19. The COVID-19 vaccine is one of the key measures to control the pandemic. However, the actual coverage of COVID-19 vaccination and associated factors remain unclear among DM patients in China. We conducted this study to investigate the COVID-19 vaccine coverage, safety, and perceptions among patients with DM in China.

**Methods:**

A cross-sectional study of a sample of 2200 DM patients from 180 tertiary hospitals in China was performed using a questionnaire developed through the Wen Juan Xing survey platform to collect information regarding their coverage, safety, and perceptions of COVID-19 vaccination. A multinomial logistic regression analysis model was performed to determine any independent relationships with COVID-19 vaccination behavior among DM patients.

**Results:**

In total, 1929 (87.7%) DM patients have received at least one dose COVID-19 vaccine, and 271 (12.3%) DM patients were unvaccinated. In addition, 65.2% (n = 1434) were booster vaccinated against COVID-19, while 16.2% (n = 357) were only fully vaccinated and 6.3% (n = 138) were only partially vaccinated. The prevalence of adverse effects after the first dose of vaccine, the second dose of vaccine, and the third dose of vaccine were 6.0%, 6.0%, and 4.3% respectively. Multinomial logistic regression analysis showed that DM patients complicated with immune and inflammatory diseases (partially vaccinated: OR = 0.12; fully vaccinated: OR = 0.11; booster vaccinated: OR = 0.28), diabetic nephropathy (partially vaccinated: OR = 0.23; fully vaccinated: OR = 0.50; booster vaccinated: OR = 0.30), and perceptions on the safety of COVID-19 vaccine (partially vaccinated: OR = 0.44; fully vaccinated: OR = 0.48; booster vaccinated: OR = 0.45) were all associated with the three of vaccination status.

**Conclusion:**

This study showed that higher proportion of COVID-19 vaccine coverage among patients with DM in China. The concern about the safety of the COVID-19 vaccine affected the vaccine behavior in patients with DM. The COVID-19 vaccine was relatively safe for DM patients due to all side effects were self-limiting.

## Introduction

The Coronavirus disease 2019 (COVID-19) vaccine plays an important role in the prevention and control of the COVID-19 epidemic. Many studies have indicated that it could effectively protect against symptomatic or asymptomatic SARS-CoV-2 infection and prevent the disease progression to severe-critical ill and death (1–3). Several variants of concern emerge in successions, such as Alpha, Beta, Gamma, Delta, and Omicron, which results in COVID-19 vaccines that based on the original strain decrease the infection protection (4), whereas COVID-19 vaccines are still effective in preventing severe or death cases against all variants of concern (5). At the time of writing (Oct 24, 2022), more than 5.41 billion people have received at least one dose of the COVID-19 vaccine worldwide, which is nearly 1.3 billion people in China and up to 91.37% of the total Chinese population (6). There is a lot of chronic diseases population in China, including diabetes mellitus (DM) (7), hypertension (8), chronic kidney disease (9), chronic obstructive pulmonary disease (10), etc. However, the vaccination coverage in these COVID-19 susceptible populations with low immunity was unclear. In the last two rounds of large-scale outbreaks in Shanghai and Hong Kong, most of the severe or fatal cases were chronic patients and most of them were not vaccinated (11, 12). Therefore, the COVID-19 vaccine shot on these fragile patients is encouraged to get protection from preventing COVID-19.

Patients with DM have a higher risk of being infected with COVID-19 and a poor prognosis after infection compared to healthy individuals (13), the possible reason is related to hyperglycemia promoting inflammatory response and coagulation abnormalities (14). Thus, patients with DM are recommended to receive the COVID-19 vaccine to increase their immunity so that protecting against infection and critical disease (15). However, some studies have observed that patients with DM had a lower vaccination intention compared to non-diabetic individuals, with a vaccine hesitation rate of 29.0% in Saudi Arabia (16), 24.7% in Malaysia (17), 56.4% in Changzhi (a city in Shan xi, China) (18). Furthermore, the actual vaccination rate of full vaccination with the second dose was 55.5% in Saudi Arabia (19). For the vaccinated coverage rate with at least one dose was 21.4% in India (20) and 31.0% in Sudan (21). The main reasons for vaccine hesitancy among DM patients are concern about the side effects and the safety of the COVID-19 vaccine (16, 18, 21, 22), although the side effects of COVID-19 vaccine have been reported to be mild and self-limiting in DM patients (23, 24). As a result, there are still obstacles for DM patients to receive the COVID-19 vaccine, which brings a great challenge to controlling the COVID-19 pandemic due to a higher prevalence of DM and a lower vaccination intention.

There were more than 158 million DM patients in China according to the latest prevalence of DM in China (25). A huge number of DM patients in China were at high risk of COVID-19 and more prone to severe or death cases. Once infected, it would lead to large consumption of medical resources if they did not get vaccinated. To date, no study has examined the prevalence of COVID-19 vaccines in patients with DM based on the Chinese population. Therefore, we carried out this cross-sectional designed study to investigate the COVID-19 actual vaccination rates. Meanwhile, the COVID-19 vaccine safety and potential factors associated with the COVID-19 vaccine status of DM patients in China were also evaluated.

## Methods

### Study design and participants

We conducted a cross-sectional online survey between 30 August to 3 October 2022. Potential participants were recruited by endocrinologists in 180 tertiary hospitals from most provinces in China, and endocrinologists would invite them to scan the QR code to access the questionnaire when they visited the department of endocrinology. The survey was administered by the biggest online survey platform Wen Juan Xing (https://www.wjx.cn). The questionnaire was designed based on the published articles that investigated the coverage and safety of the COVID-19 vaccine in China and other countries, which was reviewed by a clinically experienced endocrinologist. In addition, we also investigated the prevalence of SARS-CoV-2 infection in patients with diabetes mellitus who visit Peking Union Medical College Hospital (PUMCH) between 10 January to 10 February. Informed consent was implied by completing the online survey. This study was endorsed and conducted following the protocol approved by the Ethics Committee of PUMCH (K1965-K22C0433).

Eligible participants were as follows:1) individuals who self-reported them diagnosed with diabetes mellitus, including type 1 diabetes, type 2 diabetes, unclassified diabetes, and other specific types of diabetes; 2) Age older than 18 years; 3) voluntarily participated in the survey. Participants would be excluded if 1) the time to complete the questionnaire was less than 5 minutes; 2) answered with logically contradictory options; 3) unable to use the mobile phone to complete the questionnaire.

### Sample size calculation

Due to there is no study or report about the COVID-19 vaccination coverage of patients with DM in China, *p* = 50% to achieve the maximum sample size with a precision level of 3% (50 ± 3%) was set for this study. The sample size was determined as follows (26):


$$\frac{Z_{\alpha /2}^{2}\left(1-p\right)p}{{\text{δ}}^{2}}$$

The $Z_{\alpha /2}$ and δ was taken as 1.96 and 0.03, respectively. The sample size calculated from the formula was 1067 DM patients. After increasing about 10% invalid questionaries due to lack of information or no response, the minimum required sample size was about 1174 participants.

### Variables

The survey investigated the information included three major items: 1) sociodemographic characteristics, such as sex, age, education level, marital status, having children under age 18, work status, administrative regions, residence, and monthly personal income; 2) medical history and health status, including food or medicine allergic history, vaccine allergic history, smoking history, alcohol intake history, diabetic family history, height, weight, type of diabetes, other chronic diseases, diabetes complications, and time since diabetes diagnosis; 3) attitudes toward COVID-19 vaccine, including worried about getting COVID-19, source of COVID-19 vaccine information, believe vaccines can provide protection, perceptions on vaccine safety, factors to worry about when unvaccinated, and whether the participants consulted healthcare workers about COVID-19 vaccines injection-related problems.

Information on vaccination coverage was also collected *via* each participant who queried the vaccination information in the WeChat client applet of the State Council. All participants had to answer the question: “Have you taken COVID-19 vaccines?” (Answer options: Yes/No). If the answer was “Yes”, they further were asked to check their vaccine records before filling in the following questions. And then, they would answer the question about the time, brand, and side effects of each dose of the COVID-19 vaccine. Furthermore, the individuals who received three doses COVID-19 vaccine were investigated that their willingness to receive the fourth dose COVID-19 vaccine and their reasons for being unwilling to receive it. For unvaccinated participants, they further were asked to provide reasons for non-vaccination. Other reasons, apart from the choice options, were allowed.

### Statistical analysis

All statistical analyses were performed using SPSS v24.0 software (IBM, Armonk, NY, USA). Frequencies and proportions were used to display the categorical variables, and mean and standard deviation was used to describe the quantitative data. The Chi-square test and Fisher’s exact test were performed to preliminarily analyze various independent variables (i.e., demographics, vaccine history, vaccine knowledge, sources of information, attitudes, and beliefs) related to each of the main outcomes (vaccinated and unvaccinated). Furthermore, we divided the participants into four subgroups according to their received dose of COVID-19 vaccine, which were the unvaccinated group (not vaccinated), partially vaccinated group (received one dose), fully vaccinated group (received two doses), and booster vaccinated group (received three doses), respectively. A multinomial logistic regression analysis model was performed to determine any independent relationships with COVID-19 vaccination behavior. The dependent variable was the vaccination status (unvaccinated = 0, partially vaccinated = 1, fully vaccinated = 2, booster = 3), with the significant factors in univariate analyses between vaccinated and unvaccinated groups included as independent variables. The odds ratio (OR), 95% confidence interval (CI), and *P* -values were reported. All significance tests were two-tailed, with statistical significance set at *P*< 0.05.

## Results

### Participant characteristics

A total of 2394 participants completed the questionnaire, among them nine did not fill in their vaccination status and 185 had logically contradicted answers. Therefore, we excluded 194 invalid questionnaires and finally, 2200 valid questionnaires were included with a rate of 91.90%.

Of the 2200 participants, the ratio of male to female was around 1.3:1, and nearly one-third (n = 710, 32.3%) were elderly people (age ≥ 60). The geographic distribution shows that most of the participants came from northern China (n = 998, 45.4%) and were urban residents (n = 1870, 85.0%). More than half of participants (n = 1144, 52.0%) had high school education or below, 83.7% (n = 1841) were married or cohabiting, 39.9% (n = 878) had children under age 18, 46.5% (n = 1026) were employed, and 51.4% (n = 1129) had a monthly personal income less than 5000 yuan. In terms of medical history in included participants, 11.4% (n = 250) had a food or medicine allergic history, 3.7% (n = 82) had a vaccine allergic history, 33.9% (n = 745) had a smoking history, 31.3% (n = 688) had alcohol intake history, and 40.5% (n = 890) had a diabetic family history. Among 2200 DM patients, 85.1% (n = 1873) were type 2 diabetes, 54.4% (n = 1197) had a body mass index of more than 24.0, 36.9% (n = 812) had a DM diagnosis for over 10 years, 63.3% (n = 1393) had at least one other chronic disease, and 24.9% (n = 548) had at least one diabetes complications (**Table 1**, **2**).

**Table 1:** Sociodemographic characteristics of the study population. (n = 2200).

Characteristic	All Participants (n = 2200)	Vaccinated participants (n = 1929)	Unvaccinated participants (n = 271)	X^2^	p-value
Sex					
Male	1249 (56.8%)	1110 (57.5%)	139 (51.3%)	3.515	0.061
Female	951 (43.2%)	819 (42.5%)	132 (48.7%)		
Age group, years					
18-39	496 (22.5%)	439 (22.8%)	57 (21.0%)	37.758	**< 0.001**
40-49	406 (18.5%)	366 (19.0%)	40 (14.8%)		
50-59	588 (26.7%)	522 (27.1%)	66 (24.3%)		
60-69	475 (21.6%)	413 (21.4%)	62 (22.9%)		
70-79	191 (8.7%)	163 (8.4%)	28 (10.3%)		
≥ 80	44 (2.0%)	26 (1.4%)	18 (6.6%)		
Education level					
Below high school	661 (30.0%)	564 (29.3%)	97 (35.8%)	110.489	**< 0.001**
High school	483 (22.0%)	436 (22.6%)	47 (17.3%)		
College	948 (43.1%)	832 (43.1%)	116 (42.8%)		
Postgraduate	108 (4.9%)	937 (48.6%)	11 (4.1%)		
Marital status					
Single	217 (9.9%)	189 (9.8%)	28 (10.3%)	29.144	**< 0.001**
Married or cohabitating	1841 (83.7%)	1634 (84.7%)	207 (76.4%)		
Divorced or separated	76 (3.5%)	61 (3.2%)	15 (5.5%)		
Widowed	66 (3.0%)	45 (2.3%)	21 (7.7%)		
Have children under age 18					
Yes	878 (39.9%)	790 (40.1%)	88 (32.5%)	7.128	**0.008**
No	1322 (60.1%)	1139 (59.1%)	183 (67.5%)		
Work status					
Unemployed	281 (12.8%)	235 (12.2%)	46 (17.0%)	15.443	**0.001**
Employed	1025 (46.6%)	928 (48.1%)	97 (35.8%)		
Retired	854 (38.8%)	731 (37.9%)	123 (45.4%)		
Student	40 (1.8%)	35 (1.8%)	5 (1.8%)		
Administrative regions					
Eastern China	260 (11.8%)	228 (11.8%)	32 (11.8%)	14.45	**0.025**
Southern China	218 (9.9%)	138 (7.2%)	30 (11.1%)		
Central China	266 (12.1%)	225 (11.7%)	31 (11.4%)		
Northern China	998 (45.4%)	860 (44.6%)	138 (50.9%)		
Northwest China	113 (5.1%)	107 (5.6%)	6 (2.2%)		
Southwest Region	204 (9.3%)	187 (9.7%)	17 (6.3%)		
North East	141 (6.4%)	124 (6.4%)	17 (6.3%)		
Residence					
Rural	330 (15.0%)	292 (15.1%)	38 (14.0%)	0.232	0.63
Urban	1870 (85.0%)	1637 (84.9%)	233 (86.0%)		
Monthly personal income (Chinese yuan †)					
< 2000	250 (11.4%)	219 (11.4%)	31 (11.4%)	1.643	0.65
2000-4999	879 (40.0%)	778 (40.3%)	101 (37.3%)		
5000-10000	687 (31.2%)	602 (31.2%)	85 (31.4%)		
> 10000	384 (17.5%)	330 (17.1%)	54 (19.9%)		

p-value is from the Chi-square test.

Bold values show significant differences (P< 0.05).

**Table 2:** Medical history and health status of study participants (n = 2200).

Status	All Participants (n = 2200)	Vaccinated participants (n = 1929)	Unvaccinated participants (n = 271)	X^2^	p-value
Food or medicine allergic history					
Yes	250 (11.4%)	199 (10.3%)	51 (18.8%)	17.057	**< 0.001**
No	1950 (88.6%)	1730 (89.7%)	220 (81.2%)		
Vaccine allergic history					
Yes	82 (3.7%)	59 (3.1%)	23 (8.5%)	19.514	**< 0.001**
No	2118 (96.3%)	1870 (96.9%)	248 (91.5%)		
Smoking history					
Yes	745 (33.9%)	656 (34.0%)	89 (32.8%)	0.144	0.704
No	1455 (66.1%)	1273 (66.0%)	182 (67.2%)		
Alcohol intake history					
Yes	688 (31.3%)	623 (32.3%)	65 (24.0%)	7.637	**0.006**
No	1512 (68.7%)	1306 (67.7%)	206 (76.0%)		
Diabetic family history					
Yes	890 (40.5%)	780 (40.4%)	110 (40.6%)	0.092	0.955
No	1053 (47.9%)	925 (48.0%)	128 (47.2%)		
Uncertain	257 (11.7%)	224 (11.6%)	33 (12.2%)		
Self–reported BMI, kg/m^2^					
< 18.5	93 (4.2%)	72 (3.7%)	21 (7.7%)	12.401	**0.006**
18.5-23.9	910 (41.4%)	794 (41.1%)	116 (42.8%)		
24.0-27.9	759 (34.5%)	670 (34.7%)	89 (32.8%)		
≥ 28	438 (19.9%)	393 (20.4%)	45 (16.6%)		
Type of diabetes					
Type 1	232 (10.6%)	197 (10.2%)	35 (12.9%)	6.189	0.103
Type 2	1873 (85.1%)	1650 (85.5%)	223 (82.3%)		
Unclassified diabetes	77 (3.5%)	69 (3.6%)	8 (3.0%)		
Others	18 (0.8%)	13 (0.7%)	5 (1.8%)		
Chronic disease					
Hypertension	824 (37.5%)	701 (36.3%)	123 (45.4%)	102.818	**< 0.001**
Hyperlipidemia	817 (37.1%)	708 (36.7%)	109 (40.2%)		
Metabolic syndrome	115 (5.2%)	99 (5.1%)	16 (5.9%)		
Chronic respiratory disease	74 (3.4%)	63 (3.3%)	11 (4.1%)		
Cardiovascular and cerebrovascular diseases	258 (11.7%)	198 (10.3%)	60 (22.1%)		
Liver or kidney diseases	57 (2.6%)	43 (2.2%)	14 (5.2%)		
Inflammatory immune diseases	39 (1.8%)	22 (1.1%)	17 (6.3%)		
Cancer	50 (2.3%)	30 (1.6%)	20 (7.4%)		
Others	128 (5.8%)	100 (5.2%)	28 (10.3%)		
No	807 (36.7%)	739 (38.3%)	68 (25.1%)		
Diabetes complications					
Diabetic eye disease	280 (12.7%)	233 (12.1%)	57 (21.0%)	71.693	**< 0.001**
Diabetic Nephropathy	177 (8.1%)	129 (6.6%)	49 (18.1%)		
Diabetic foot	65 (3.0%)	45 (2.3%)	20 (7.4%)		
Diabetic neuropathy	290 (13.2%)	235 (12.2%)	54 (19.9%)		
No	1652 (75.1%)	1479 (76.7%)	172 (63.5%)		
Time since diabetes diagnosis, years					
≤1	499 (22.7%)	456 (23.6%)	43 (15.9%)	15.38	**< 0.001**
1–10	889 (40.4%)	788 (40.9%)	101 (37.3%)		
≧10	812 (36.9%)	685 (35.5%)	127 (46.9%)		

p-value is from the Chi-square test.

Bold values show significant differences (P< 0.05).

a This was a multiple-choice question.

### Attitudes and beliefs toward the COVID-19 vaccine

More than half of DM patients (n = 1222, 55.5%) were worried about getting COVID-19. 31.4% (n = 690) learned about the information of COVID-19 vaccine from the Internet, followed by communication with friends and family (n = 469,21.3%), consult with healthcare workers (n = 460, 20.9%), epidemic prevention station (n = 405, 18.4%), and other sources (n = 176, 8.0%). A majority (n = 1943, 88.3%) of participants believed that the COVID-19 vaccine could provide protection. Meanwhile, 73.3% (n = 1613) of participants thought that the COVID-19 vaccines were safe or very safe (**Table 3**).

**Table 3:** Attitudes toward COVID-19 vaccine among patients with DM in China.

Variables	All Participants (n = 2200)	Vaccinated participants (n = 1929)	Unvaccinated participants (n = 271)	X^2^	p-value
Have ever got COVID-19					
Yes	11 (0.5%)	10 (0.5%)	1 (0.4%)	0.107	0.744^a^
No	2189 (99.5%)	1919 (99.5%)	270 (99.6%)		
Worried about getting COVID-19					
Yes	1222 (55.5%)	1073 (55.6%)	149 (55.0%)	0.04	0.842
No	978 (44.5%)	856 (44.4%)	122 (45.0%)		
Source of COVID-19 vaccine information					
Internet	690 (31.4%)	588 (30.5%)	102 (37.6%)	23.45	**< 0.001**
Communication with friends and family	469 (21.3%)	400 (20.7%)	69 (25.5%)		
Consult with healthcare workers	460 (20.9%)	407 (21.1%)	53 (19.6%)		
Epidemic prevention station	405 (18.4%)	382 (19.8%)	23 (8.5%)		
Others	176 (8.0%)	152 (7.9%)	24 (8.9%)		
Believe vaccines can provide protection					
Yes	1943 (88.3%)	1740 (90.2%)	203 (74.9%)	53.875	**< 0.001**
No	257 (11.7%)	189 (9.9%)	68 (25.1%)		
Perceptions on vaccine safety					
Very Safe	570 (25.9%)	526 (27.3%)	44 (16.2%)	84.71	**< 0.001**
Safe	1043 (47.4%)	943 (48.9%)	100 (36.9%)		
General	506 (23.0%)	407 (21.1%)	99 (36.5%)		
Unsafe	65 (3.0%)	40 (2.1%)	25 (9.2%)		
Very unsafe	16 (0.7%)	13 (0.7%)	3 (1.1%)		

a p-value for Fisher’s exact test, others for the Chi-square test.

Bold values show significant differences (P< 0.05).

### COVID-19 vaccination status in patients with diabetes

**Figure 1:**
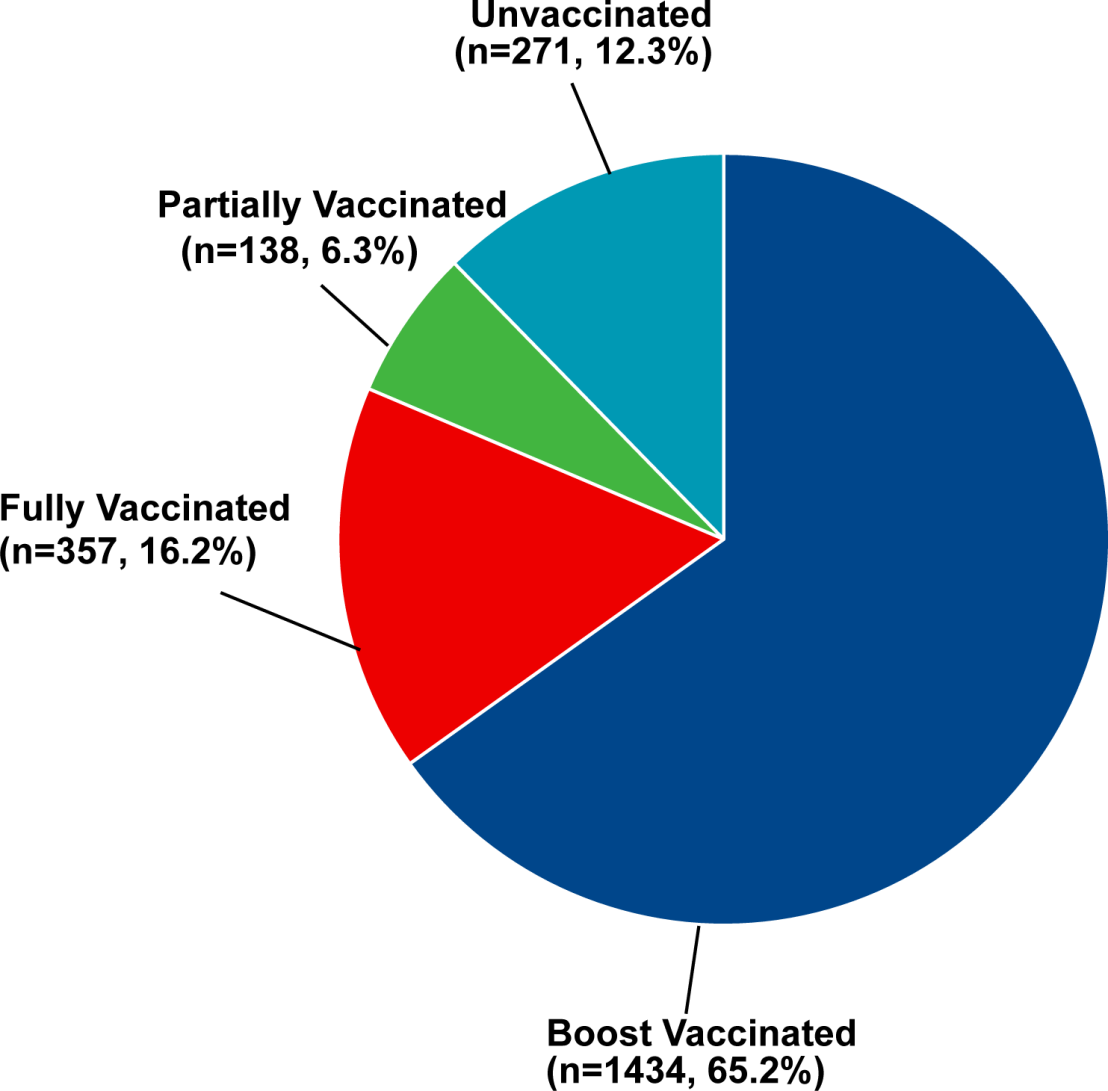
The COVID-19 vaccination status among patients with DM (n = 2200).

The coverage rate of the COVID-19 vaccine in survey participants was 87.7% (n = 1929). Moreover, 65.2% (n = 1434) were booster vaccinated (received three doses of COVID-19 vaccine) against COVID-19, while 16.2% (n = 357) were only fully vaccinated (received only two doses of COVID-19 vaccine) and 6.3% (n = 138) were only partially vaccinated (received one dose COVID-19 vaccine) (**Figure 1**). Inactivated vaccines, including Sinopharm, Sinovac, KCONVAC, and IMBCAMS were used in 91.8%, 91.6%, and 86.1% of the first, second, and third dose of vaccines, respectively. Sinovac was the most common vaccine type, representing nearly half of each dose. In contrast, the viral vector-based vaccine (CanSinoBio), and protein subunit vaccine (Zhifei Longcom) were used on a relatively small scale (**Table 4**).

**Table 4:** COVID-19 vaccine type and safety data of vaccinated patients with DM, stratified by COVID-19 vaccinated dose.

Variables	The First dose vaccine (n=1929)	The second dose vaccine (n=1791)	The third dose vaccine (n=1434)
Vaccine manufacturer			
Sinopharm	785 (40.7%)	702 (39.2%)	521 (36.3%)
Sinovac	973 (50.4%)	929 (51.9%)	706 (49.2%)
CanSinoBIO	11 (0.6%)	6 (0.3%)	7 (0.5%)
Zhifei Longcom	67 (3.5%)	62 (3.5%)	84 (5.9%)
KCONVAC	10 (0.5%)	7 (0.4%)	5 (0.3%)
IMBCAMS	3 (0.2%)	2 (0.1%)	4 (0.3%)
Others/Uncertain	80 (4.1%)	83 (4.6%)	107 (7.5%)
Safety			
Side effects after vaccine (n, %)	115 (6.0%)	108 (6.0%)	62 (4.3%)
Timing of onset, days (median)	1 (1,3)	2 (1,3)	2 (1,2)
Adverse effects			
Fatigue	57 (3.0%)	31 (1.7%)	25 (1.7%)
Sleepless	31 (1.6%)	9 (0.5%)	7 (0.5%)
Fever	19 (1.0%)	15 (0.8%)	6 (0.4%)
Muscle soreness	33 (1.7%)	26 (1.5%)	14 (1.0%)
Local pain	14 (0.7%)	19 (1.1%)	14 (1.0%)
Headache	15 (0.8%)	11 (0.6%)	7 (0.5%)
Nausea/vomiting	16 (0.8%)	4 (0.2%)	2 (0.1%)
Pruritus	19 (1.0%)	9 (0.5%)	8 (0.6%)
Arthralgia	17 (0.9%)	9 (0.5%)	6 (0.4%)
diarrhoea	1 (0.5%)	3 (0.2%)	0 (0%)
Others	33 (1.7%)	22 (1.2%)	16 (1.1%)
**Self-reported severe adverse effects**	0	0	0

### Adverse effects of COVID-19 vaccine in patients with diabetes

The side effects of each dose COVID-19 vaccine in DM patients after injection were investigated in this study. Among DM patients who received the first dose COVID-19 vaccine, 6.0% (n = 115) of patients reported experiencing adverse events (**Table 4**). Fatigue (n = 57, 3.0%) was the most reported adverse effect after receiving the first dose COVID-19 vaccine, followed by muscle soreness (n = 33, 1.7%) and sleeplessness (n = 31, 1.6%). The median time from the first vaccination shot to the onset of adverse effects was one day. The prevalence of adverse effects after the second dose of vaccine and the third dose of the vaccine was 6.0% and 4.3% respectively. Fatigue was also the most reported adverse effect for those participants who received the second (n = 31, 1.7%) or third dose (n = 25, 1.7%) COVID-19 vaccine. Both median times from vaccination shot to onset of adverse effects was 2 days. All of the adverse effects were mild to moderate and self-relieving. No severe adverse effects were observed in these included participants.

### Reason to not receive COVID-19 vaccine

**Figure 2:**
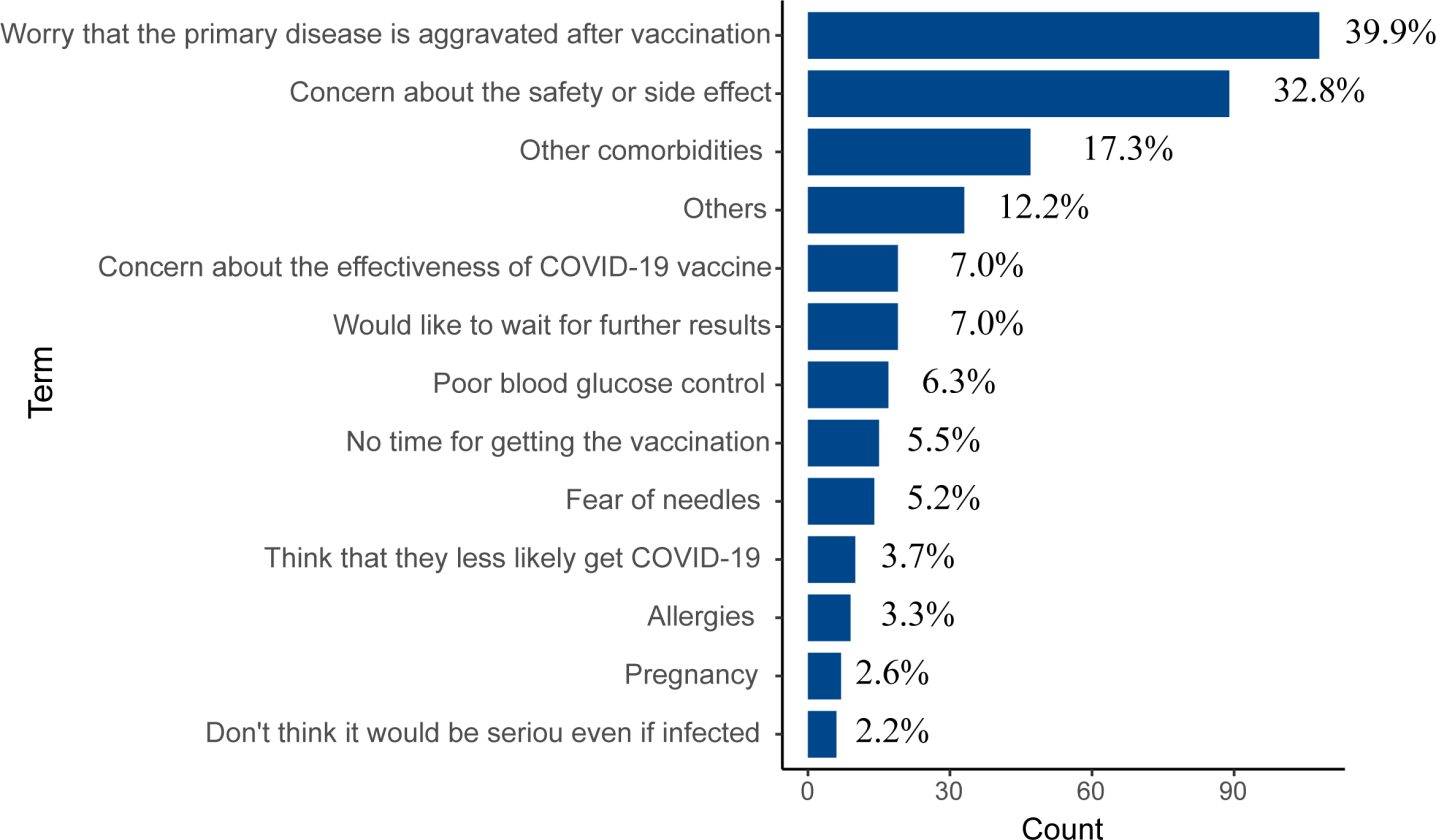
The reasons for not receiving COVID-19 vaccines (n = 271).

Two hundred and seventy-one (12.3%) have never received any COVID-19 vaccine. The reasons for non-vaccination were shown in **Figure 2**. The top two reasons were concerning about the primary disease worsening after vaccination (n = 108, 39.9%) and the safety or side effects of the COVID-19 vaccine (n = 89, 32.8%). 17.3% (n = 47) had other comorbidities that lead to unsuitable for vaccination, and 14.0% (n = 38) worried that the effectiveness of the COVID-19 vaccine or waiting for further results from other vaccinated individuals. Furthermore, there were some medical reasons, such as poor blood glucose control (n = 17, 6.3%), fear of needles (n = 14, 5.2%), allergies (n = 9, 3.3%), and preparation for pregnancy (n = 7, 2.6%). Besides, some participants (n = 15, 5.5%) claimed that they did not have time to get the vaccination, 3.7% (n = 10) thought that they were less likely to get COVID-19, and 2.2% (n = 6) did not think it would be serious even if infected.

### Factors associated with COVID-19 vaccination behavior in patients with diabetes

In this study, we analyzed the factors associated with COVID-19 vaccination behavior in patients with DM by using a multinomial logistic regression model, which could estimate the odds ratio of DM patients being partially vaccinated, fully vaccinated, or booster vaccinated versus unvaccinated. We selected 16 statistically significant factors from the univariate analysis in sociodemographic characteristics, medical history, health status, attitudes and beliefs toward the COVID-19 vaccine of DM patients, entering into a multinomial logistic regression model (**Table 5**).

**Table 5:** Factors associated with COVID-19 vaccination status.

Factors	Partially Vaccinated			Fully Vaccinated			Booster Vaccinated		
	OR	95% CI	*P*	OR	95% CI	*P*	OR	95% CI	*P*
Age group, years									
18-39	Ref			Ref			Ref		
40-49	0.78	0.36-1.70	0.533	1.1	0.61-1.99	0.746	1.43	0.85-2.40	0.176
50-59	1.16	0.54-2.51	0.699	0.91	0.49-1.71	0.772	1.39	0.81-2.39	0.227
60-69	1.17	0.43-3.23	0.756	1.46	0.64-3.31	0.369	1.25	0.62-2.50	0.538
70-79	1.03	0.31-3.46	0.958	1.59	0.61-4.15	0.344	1.42	0.63-3.21	0.394
≥ 80	0.59	0.12-2.91	0.520	0.40	0.10-1.55	0.183	0.33	0.12-0.94	0.039
Education level									
Below high school	Ref			Ref			Ref		
High school	1.47	0.81-2.65	0.204	1.55	0.94-2.54	0.085	1.47	0.96-2.24	0.077
College	0.86	0.49-1.51	0.596	1.39	0.89-2.16	0.147	1.31	0.90-1.91	0.155
Postgraduate	0.97	0.31-3.03	0.961	1.25	0.52-3.00	0.623	1.36	0.64-2.89	0.432
Marital status									
Single	Ref			Ref			Ref		
Married or cohabitating	0.63	0.28-1.44	0.277	0.81	0.42-1.58	0.54	1.05	0.58-1.88	0.877
Divorced or separated	0.45	0.11-1.87	0.273	0.63	0.22-1.82	0.634	0.83	0.34-2.04	0.688
Widowed	0.34	0.08-1.44	0.142	0.22	0.07-0.78	0.018	0.63	0.26-1.54	0.312
Have children under age 18									
Yes	1.41	0.85-2.32	0.183	1.33	0.89-1.98	0.166	1.19	0.84-1.67	0.330
No	Ref			Ref			Ref		
Work status									
Unemployed	Ref			Ref			Ref		
Employed	1.41	0.71-2.83	0.331	1.60	0.91-2.82	0.103	1.66	1.04-2.67	**0.036**
Retired	1.22	0.54-2.79	0.631	1.34	0.68-2.63	0.404	1.6	0.91-2.80	0.102
Student	0.42	0.04-4.38	0.465	3.38	0.94-12.19	0.063	1.39	0.41-4.76	0.600
Administrative regions									
Eastern China	Ref			Ref			Ref		
Southern China	1.16	0.44-3.02	0.765	1.66	0.80-3.45	0.177	0.94	0.51-1.74	0.844
Central China	1.28	0.52-3.17	0.592	1.7	0.83-3.46	0.145	1.00	0.55-1.83	0.99
Northern China	1.14	0.54-2.39	0.733	1.22	0.68-2.18	0.511	0.87	0.54-1.40	0.554
Northwest China	2.22	0.60-8.29	0.235	3.26	1.11-9.57	**0.032**	1.89	0.71-5.00	0.202
Southwest Region	2.45	0.89-6.76	0.084	1.97	0.85-4.56	0.114	1.92	0.96-3.85	0.066
North East	1.10	0.37-3.25	0.875	1.26	0.53-3.00	0.600	0.94	0.46-1.95	0.871
Food or medicine allergic history									
Yes	1.03	0.54-1.95	0.938	0.79	0.47-1.32	0.360	0.72	0.47-1.10	0.126
No	Ref			Ref			Ref		
Vaccine allergic history									
Yes	0.23	0.05-1.05	0.058	0.66	0.30-1.43	0.288	0.63	0.33-1.20	0.159
No	Ref			Ref			Ref		
Alcohol intake history									
Yes	1.18	0.71-1.94	0.523	1.08	0.73-1.62	0.672	1.44	1.03-2.02	**0.036**
No	Ref			Ref			Ref		
Self–reported BMI, kg/m^2^									
< 18.5	Ref			Ref			Ref		
18.5-23.9	1.42	0.54-3.78	0.478	1.83	0.87-3.84	0.112	2.33	1.24-4.35	**0.008**
24.0-27.9	1.75	0.64-4.77	0.273	1.70	0.79-3.69	0.178	2.72	1.43-5.21	**0.002**
≥ 28	2.01	0.70-5.76	0.193	2.18	0.96-4.93	0.062	2.91	1.46-5.83	**0.003**
Other chronic diseases									
Hypertension									
Yes	0.78	0.44-1.39	0.401	0.86	0.53-1.37	0.517	0.96	0.65-1.41	0.826
No	Ref			Ref			Ref		
Hyperlipidemia									
Yes	1.18	0.67-2.07	0.569	0.83	0.53-1.37	0.400	1.23	0.85-1.78	0.275
No	Ref			Ref			Ref		
Metabolic syndrome									
Yes	1.67	0.62-4.6	0.308	1.50	0.55-4.10	0.433	1.50	0.75-2.99	0.250
No	Ref			Ref			Ref		
Chronic respiratory disease									
Yes	1.93	0.61-6.13	0.266	1.49	0.54-4.07	0.442	1.85	0.80-4.29	0.152
No	Ref			Ref			Ref		
Cardiovascular and cerebrovascular diseases									
Yes	0.75	0.39-1.45	0.389	1.03	0.62-1.71	0.923	0.52	0.33-0.80	**0.003**
No	Ref			Ref			Ref		
Liver and kidney diseases									
Yes	0.83	0.21-3.35	0.793	0.96	0.35-2.59	0.930	0.96	0.42-2.21	0.963
No	Ref			Ref			Ref		
Inflammatory immune diseases									
Yes	0.12	0.02-0.99	**0.049**	0.11	0.02-0.51	**0.005**	0.28	0.12-0.64	**0.002**
No	Ref			Ref			Ref		
Cancer									
Yes	0.52	0.16-1.73	0.288	0.19	0.06-0.61	**0.005**	0.25	0.12-0.51	**< 0.001**
No	Ref			Ref			Ref		
Others									
Yes	0.43	0.16-1.16	0.094	0.4	0.19-0.85	**0.016**	0.52	0.29-0.92	**0.024**
No	Ref			Ref			Ref		
None									
Yes	1.02	0.49-2.12	0.97	1.2	0.67-2.14	0.545	1.3	0.80-2.12	0.293
No	Ref			Ref			Ref		
Diabetes complications									
Diabetic eye disease									
Yes	1.10	0.48-2.55	0.819	1.01	0.53-1.94	0.98	0.59	0.34-1.01	0.052
No	Ref			Ref			Ref		
Diabetic Nephropathy									
Yes	0.23	0.09-0.58	**0.002**	0.51	0.26-0.99	**0.048**	0.3	0.17-0.53	**< 0.001**
No	Ref			Ref			Ref		
Diabetic foot									
Yes	0.51	0.15-1.76	0.286	0.82	0.34-1.98	0.658	0.39	0.18-0.83	**0.014**
No	Ref			Ref			Ref		
Diabetic neuropathy									
Yes	0.92	0.40-2.12	0.844	0.75	0.39-1.44	0.388	0.71	0.41-1.22	0.214
No	Ref			Ref			Ref		
None									
Yes	0.66	0.25-1.74	0.397	0.89	0.42-1.89	0.763	0.58	0.31-1.08	0.086
No	Ref			Ref			Ref		
Time since diabetes diagnosis, years									
≤1	Ref			Ref			Ref		
1–10	0.6	0.33-1.11	0.102	0.67	0.42-1.08	0.103	0.86	0.56-1.32	0.489
≧10	0.78	0.41-1.51	0.466	0.67	0.39-1.13	0.131	0.9	0.57-1.42	0.659
Source of COVID-19 vaccine information									
Internet	0.49	0.22-1.10	0.082	0.86	0.43-1.70	0.655	0.70	0.40-1.23	0.211
Communication with friends and family	0.62	0.26-1.45	0.266	1.14	0.56-2.35	0.717	0.79	0.44-1.42	0.424
Consult with healthcare workers	0.75	0.32-1.76	0.505	1.34	0.65-2.77	0.433	0.83	0.46-1.51	0.544
Epidemic prevention station	1.73	0.70-4.28	0.232	2.57	1.16-5.69	**0.020**	1.93	0.98-3.79	0.057
Others	Ref			Ref			Ref		
Believe COVID-19 vaccines can provide protection									
Yes	1.69	0.80-3.59	0.232	1.12	0.67-1.89	0.662	2.08	1.34-3.22	**0.001**
No	Ref			Ref			Ref		
Perceptions on vaccine safety									
Very Safe	Ref			Ref			Ref		
Safe	0.89	0.51-1.56	0.688	1.01	0.63-1.61	0.978	0.83	0.55-1.24	0.353
General	0.44	0.22-0.87	**0.018**	0.48	0.28-0.82	**0.007**	0.45	0.29-0.70	**< 0.001**
Unsafe	0.34	0.09-1.27	**0.11**	0.74	0.32-1.71	0.480	0.08	0.03-0.20	**< 0.001**
Very unsafe	0.49	0.04-5.62	0.566	0.76	0.15-3.76	0.731	0.34	0.08-1.54	0.160

p-value is from a multinomial logistic regression analysis model.

Bold values show significant differences (P< 0.05).

The age was not found to be associated with being partially vaccinated or fully vaccinated compared to those not being vaccinated, while DM patients whose age was more than 80 years compared to those aged 18-39 years were found to be less likely to be booster vaccinated compared to those not being vaccinated (OR = 0.33, 95% CI: 0.12-0.94). The widowed participants were 78% less likely to be fully vaccinated compared to those single (OR = 0.22, 95% CI: 0.06-0.78). The employed participants were observed to have 1.65 times higher chances of being booster vaccinated compared to those unvaccinated (OR = 1.66, 95% CI: 1.04-2.67). Compared to participants who lived in Eastern China, the DM patients who lived in Northwest China were 3.26 times more likely to be fully vaccinated compared with those unvaccinated (OR = 3.26, 95% CI: 1.11-9.57).

With respect to the medical history and health status, those DM patients who had an alcohol intake history when compared to those without had 1.44 times higher chances of being booster vaccinated compared to unvaccinated (OR = 1.44, 95% CI: 1.03-2.02). Compared to DM patients with BMI less than 18.5, those whose BMI was between 18.5 and 23.9 were 2.33 times more likely to be booster vaccinated (OR =2.33, 95% CI: 1.24-4.35), those whose BMI was between 24.0 and 27.9 were 2.72 times more likely to be booster vaccinated (OR =2.72, 95% CI: 1.43-5.21), and those whose BMI was more than 28.0 were 2.90 times more likely to received three doses of COVID-19 vaccine (OR =2.91, 95% CI: 1.46-5.83). DM patients with immune and inflammatory diseases were 88% less likely to be partially vaccinated (OR = 0.12, 95% CI: 0.02-0.99), 89% less likely to be fully vaccinated (OR = 0.11, 95% CI: 0.02-0.51), and 72% less likely to be booster vaccinated (OR = 0.28, 95% CI: 0.12-0.64). In addition, DM patients with cancer were 81% less likely to be fully vaccinated (OR = 0.19, 95% CI: 0.06-0.61), and 75% less likely to be booster vaccinated (OR = 0.25, 95% CI: 0.12-0.51). Furthermore, DM patients with diabetic nephropathy were 77% less likely to be partially vaccinated (OR = 0.23, 95% CI: 0.09-0.58), 49% less likely to be fully vaccinated (OR = 0.51, 95% CI: 0.26-0.99), and 70% less likely to be booster vaccinated (OR = 0.30, 95% CI: 0.17-0.53) compared to those who were unvaccinated. Meanwhile, DM patients with diabetic foot were 61% less likely to be booster vaccinated compared to those not vaccinated (OR = 0.39, 95% CI: 0.18-0.83).

In terms of attitudes and beliefs toward the COVID-19 vaccine, those who got information from epidemic prevention station were 2.55 times higher chances of being fully vaccinated compared to those who were unvaccinated (OR = 2.57, 95% CI: 1.16-5.59). Patients with DM who believe COVID-19 vaccines can provide protection had 2.08 times higher chance of being booster vaccinated compared to those who were unvaccinated (OR = 2.08, 95% CI: 1.34-3.22). Compared with DM patients who thought the COVID-19 vaccine very safe, the likelihood of booster vaccinated was 55% lower among those who had a neutral attitude (OR = 0.45, 95% CI: 0.29-0.70), 92% lower among those who thought unsafe (OR = 0.08, 95% CI: 0.03-0.20). Meanwhile, those who had a neutral attitude toward the COVID-19 vaccine were 56% less likely to be partially vaccinated (OR = 0.44, 95% CI: 0.22-0.87) and 52% less likely to be fully vaccinated compared to those who thought very safe (OR = 0.48, 95% CI: 0.28-0.82).

### Willingness to receive the fourth dose of the COVID-19 vaccine

**Figure 3:**
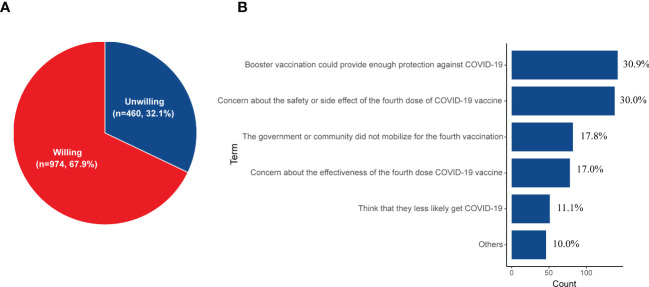
**(A)** The willingness for receiving the fourth dose of the COVID-19 vaccine among those who have finished the booster vaccination (n = 1434). **(B)** The reasons for being unwilling to receive the fourth dose of the COVID-19 vaccine (n = 460).

Participants’ willingness to receive the fourth dose of the COVID-19 vaccine was explored among 1434 booster vaccinated cases. Of the 1434 DM patients, 67.9% (n = 974) would willing to receive the fourth dose of the COVID-19 vaccine (**Figure 3A**). Participants’ reasons for not taking the fourth dose COVID-19 vaccine are illustrated in **Figure 3B**. The most common reason was “Boost vaccination could provide enough protection against COVID-19” (n = 143, 30.9%), followed by the notion that “concern about the safety or side effect of the fourth dose COVID-19 vaccine” (n = 138, 30.0%). Some DM patients (n = 82, 17.8%) thought that the government or community did not mobilize for the fourth vaccination, so they did not want to take the next dose. In addition, 17.0% (n = 78) were concerned about the effectiveness of the fourth dose COVID-19 vaccine and 11.1% (n = 51) thought that they were less likely to get COVID-19.

### Prevalence and symptoms of SARS-CoV-2 infection in patients with DM

In early December 2022, the Chinese government optimized the policy of epidemic prevention and control such as shortening the isolation duration and reducing the frequency of SARS-CoV-2 PCR tests. Therefore, SARS-CoV-2 has widely spread in China. Two hundred DM patients have investigated the prevalence of SARS-CoV-2 infection. We observed that 179 DM patients (89.5%) were infected with SARS-CoV-2. The clinical characteristics of infected participants and non-participants are shown in **Table 6**. Furthermore, we investigated the symptoms of SAS-COV-2 infection in patients with DM. As shown in **Table S1**, the most common symptoms of SARS-CoV-2 infection were fever (n = 151, 84.3%), followed by fatigue (n = 104, 58.1%), cough (n = 102, 57.0%), and so on. In addition, it was noted that the fasting blood glucose of 30.7% (n = 55) DM patients increased after getting the SARS-CoV-2 infection.

**Table 6:** Clinical characteristics of the SARS-CoV-2 infected and uninfected patients (n = 200).

Characteristic	SARS-CoV-2 infected participants (n = 179)	SARS-CoV-2 uninfected participants (n = 21)	X^2^	p-value
Sex				
Male	95 (53.1%)	12 (57.1%)	0.125	0.724
Female	84 (46.9%)	9 (42.9%)		
Age group, years				
< 60	120 (67.0%)	8 (38.1%)	6.834	**0.009**
≥ 60	59 (33.0%)	13 (61.9%)		
Type of diabetes				
Type 1	15 (8.4%)	1 (4.8%)	0.757	0.86^a^
Type 2	147 (82.1%)	18 (85.7%)		
Unclassified diabetes	14 (7.8%)	2 (9.5%)		
Others	3 (1.7%)	0		
Chronic disease				
Hypertension	68 (38.0%)	8 (38.1%)	0.00009	0.992
Hyperlipidemia	91 (50.8%)	12 (57.1%)	0.299	0.584
Metabolic syndrome	5 (2.8%)	0	0.602	1 ^a^
Chronic respiratory disease	9 (5.0%)	0	1.106	0.602 ^a^
Cardiovascular and cerebrovascular diseases	33 (18.4%)	0	4.637	**0.028** ^a^
Liver or kidney diseases	15 (8.4%)	1 (4.8%)	0.334	1 ^a^
Inflammatory immune diseases	3 (1.7%)	1 (4.8%)	0.913	0.361 ^a^
Cancer	8 (4.5%)	0	0.978	1 ^a^
Others	6 (3.4%)	2 (9.5%)	1.864	0.2 ^a^
No	49 (27.4%)	6 (28.6%)	0.014	0.907
Diabetes complications				
Diabetic eye disease	26 (14.5%)	1 (4.8%)	1.534	0.32 ^a^
Diabetic Nephropathy	13 (7.3%)	1 (4.8%)	0.181	1 ^a^
Diabetic foot	2 (1.1%)	0	0.237	1 ^a^
Diabetic neuropathy	11 (6.1%)	0	1.366	0.61 ^a^
No	137 (76.5%)	19 (90.5%)	2.128	0.174
Time since diabetes diagnosis, years				
≤1	20 (11.2%)	0	3.523	0.172 ^a^
1–10	74 (41.3%)	12 (57.1%)		
≧10	85 (47.5%)	9 (42.9%)		
COVID-19 vaccination status				
Unvaccinated	39 (21.8%)	4 (19.0%)	6.32	0.176 ^a^
One vaccine dose	2 (1.1%)	1 (4.8%)		
Two vaccine doses	23 (12.8%)	0		
Three vaccine doses	109 (60.9%)	14 (66.7%)		
Four vaccine doses	6 (3.4%)	2 (9.6%)		
Cohabitant infected with SARS-COV-2				
Yes	163 (91.1%)	12 (57.1%)	19.77	**< 0.001**
No	16 (8.9%)	9 (42.9%)		

a p-value for Fisher’s exact test, others for the Chi-square test.

Bold values show significant differences (P< 0.05).

## Discussion

In this study, the majority of DM patients have received at least one dose COVID-19 vaccine (n = 1929, 87.7%), 81.4% (n = 1791) have received two doses of COVID-19 vaccine and 65.2% (n = 1434) have received three doses of COVID-19 vaccine. However, there were still 12.3% of DM patients (n = 271) who have never taken any COVID-19 vaccine. Up to July 23, 2022, nationwide, the coverage rate of the first dose of the COVID-19 vaccine is 92.1%, the fully vaccinated coverage rate is 89.7%, and the booster vaccinated coverage rate is 71.7% according to the report of the National Health Commission of the Peoples’s Republic of China (27). The COVID-19 vaccine coverage rate among DM patients was lower than the general population in China, while this difference was not dramatic due to the overall vaccination rate being only about 5% less than that of the whole country. The potential factors may be related to the publicity by the government and the DM patients with good glycemic control recommended by clinicians in China. Therefore, the majority of DM patients were willing to receive the COVID-19 vaccine in this real-world study. In addition, our studies showed a higher actual vaccine coverage with at least one dose in Chinese DM patients (87.7%) compared with the study in India (21.4%) and Sudan (31.0%). The potential reason might be associated with the later investigation time in this study compared with others and the shortage of vaccination supply in India (28) and Sudan (29).

Previous studies have investigated the willingness to receive the COVID-19 vaccine in patients with DM from Saudi Arabia, Changzhi, and Italy, which indicated that 14.2%-29.0% of patients with DM refused to uptake the vaccine (16, 18, 30, 31). Two hundred and seventy-one DM patients (12.3%) were still unvaccinated when we carried out this survey, the prevalence of non-vaccination in this study is relatively lower compared to other studies that reported the prevalence of COVID-19 vaccine hesitancy. The main reason for unvaccinated participants was worried the primary disease would aggravate after vaccination, followed by concern about the safety or side effects and other chronic diseases. The top two major reasons for being unvaccinated were associated with lower confidence in the safety of the COVID-19 vaccine. We also observed that a higher proportion of unvaccinated DM patients thought that the COVID-19 vaccine was unsafe or very unsafe compared to those vaccinated (10.3% vs 2.8%, *P* < 0.001). The data on COVID-19 inactivated vaccine safety on chronic diseases was insufficient, which may result in the minority of DM patients remaining unvaccinated. DM patients often have other chronic diseases, including hypertension, cardiovascular disease, kidney disease, etc (32). In this study, 63.3% (n = 1393) of participants had reported that they had at least one kind of disease beside DM. Moreover, the prevalence of other chronic diseases in unvaccinated patients with DM was higher compared to those vaccinated (74.9% vs 61.7%, *P* < 0.001). Some DM patients were not suitable for vaccination due to not getting effective control of their other diseases or comorbidities, which also impeded the COVID-19 vaccine taking in DM patients.

The side effects of the COVID-19 vaccine have not been investigated on DM patients in China. Most of the DM patients in this study received the inactivated vaccine in each dose, 91.7% for the first dose, 91.5% for the second dose, and 86.1% for the third dose, respectively. Solicited injection-site pain, fatigue, and headache were the most common adverse effects after receiving inactivated COVID-19 vaccines in healthy individuals (33). In this study, we also observed that fatigue was the most common adverse effect in patients with DM, which is in line with the results of the phase 3 trial about inactivated COVID-19 vaccines (34). Furthermore, Xiang et al. also observed that fatigue (2.2% (2/89)) was the most common adverse event in DM patients who received the second dose of inactivated vaccine (35), Dechates et al. found that fatigue (11.1% (3/27)) was common side effects in DM patients who finished the full vaccination (24). However, the sample size was small and the adverse effects of DM patients who received partial and booster vaccination were not evaluated in these studies. In addition, several studies have investigated the safety of inactivated COVID-19 vaccine in patients, including liver disease (36, 37), autoimmune inflammatory rheumatic diseases (38, 39), cancer (40), etc. Most of the adverse effects of these diseases were self-limited and moderate, while severe adverse effects were rare. Our study also found a minority of DM patients reported a lower prevalence of adverse effects with nearly 4.3%-6.1% in each dose compared to other studies in immune-related diseases and liver diseases. No DM reported that they had severe adverse effects that need to be hospitalized or fatal adverse effects, which indicated that COVID-19 vaccines were safe according to our data.

To improve the effectiveness of the vaccine against COVID-19, it is recommended to booster vaccination in those who finished the full vaccination. In China, the individuals who received two doses of inactivated COVID-19 vaccine needed to uptake the third dose vaccine to finish the booster vaccination. Several factors were found to be associated with the real vaccination status in patients with DM. We defined the DM patients who received only one dose COVID-19 vaccine were partially vaccinated, only two doses were fully vaccinated, and three doses were booster vaccinated. Patients aged more than 80 years old are less likely to finish booster vaccination. These elderly DM patients may have more contraindications to vaccinations and less opportunity to go out which reduces the chance of infection. Employed DM patients were also found to be associated with receiving three doses of the COVID-19 vaccine compared to those unemployed. In China, the government promoted eligible vaccination access to the COVID-19 vaccine among individuals who work with high infection risks, such as healthcare workers, logistics personnel, customs border inspectors, etc (41). This policy has increased the proportion of vaccination in employed DM patients. Additionally, the survey performed among a representative sample of the Australian population found that employed individuals were more willing to receive the COVID-19 vaccine. Some comorbidities of patients with DM resulted in a lower likelihood of receiving the COVID-19 vaccine compared to those without, especially in DM patients with inflammatory immune diseases or diabetic nephropathy. Several studies indicated that patients with immune-related diseases were more unwilling to receive the COVID-19 vaccine compared to the general population (42, 43). The effectiveness of the patients with the immune-mediated inflammatory disease was limited due to immunosuppressive therapies (44). Diabetic nephropathy is the independent risk factor for the poor prognosis of COVID-19 (45). The vaccination of these immunocompromised populations should be a concern. DM patients who believe that the COVID-19 vaccine could provide protection were more likely to accept the booster vaccination. In terms of perceptions of vaccine safety, DM patients who thought the safety of vaccines were general were more unlikely to receive them compared to those who thought were very safe. The concern about the safety of the COVID-19 vaccine affected the vaccine behavior. Inadequate awareness of COVID-19 vaccine safety may lead to unwillingness to receive (46, 47). The governmental recommendation is one of the important means to persuade people to receive the COVID-19 vaccine (48), more efforts should be concentrated on propagating the effectiveness and safety of the COVID-19 vaccine. In this study, we found that the higher the BMI, the more likely to complete the booster vaccination. A population-based cohort study in England also found that people who had underweight (BMI<18.5) were less likely to receive three doses of COVID-19 vaccines, while those whose BMI of more than 18.5 had a higher proportion of booster vaccination compared to those who had underweight (49). Elevated BMI is associated with poor outcomes and mortality from COVID-19 (50, 51). Meanwhile, some studies observed that the immunogenicity of the COVID-19 vaccine was reduced in obese individuals (52, 53). Therefore, it is necessary for diabetes patients to control their weight to resist infection.

The constant emergence of SARS-COV-2 variants further increases the risk of transmission (54), which leads to a growing number of COVID-19 patients. Therefore, many countries have approved the fourth dose of the COVID-19 vaccine (55–57). Up to the writing of this article, the fourth dose of the vaccine has not been widely promoted in China, but it might be approved in the not too distant future (58). Therefore, we have investigated the willingness to receive the fourth dose COVID-19 vaccine in those who had received three doses of the vaccine. A relatively low acceptance of the fourth dose in DM patients who were booster vaccinated was observed. A majority of those who were hesitant thought the third dose could provide protection or were concerned about the safety of the fourth dose. Nearly half of DM patients (48.7%, n = 1034) in this study have passed more than 6 months from those who received the third dose of vaccine to the date that they filled in the questionnaire. The immunogenicity of the third vaccine dose would be winning with the time extension after vaccination (59). Although these patients with DM have received three doses of vaccine, the efficacy of the vaccine has weakened over time, and they still have a high risk of infection with COVID-19. In addition, some participants indicated that the government or community did not require the fourth vaccination, which lead to their willingness for the fourth dose decreased. Therefore, it is possible to increase the vaccination rate depending on the function of the government and endocrinologists in improving awareness and eliminating false perceptions of COVID-19 vaccines among DM patients.

On December 7, 2022, the Chinese government optimized COVID-19 policies, such as public places no longer requiring proof of negative nucleic acid testing results or checking digital health codes of visitors and cross-regional travelers were not isolation needed if they could not provide negative nucleic acid testing results (60). Our study showed that nearly 89.5% (n = 179) of DM patients got SARS-CoV-2 infection in December 2022. The age of infected DM patients was younger than those uninfected (*P* = 0.009, **Table 6**), which was associated with more needs for social contact for work, commuting, shopping, and other in young DM patients compared to those elder. Meanwhile, the prevalence of close contacts and co-residents of infected patients getting infection had a higher proportion compared to those uninfected patients (*P*< 0.001, **Table 6**), indicating that the chances of contact with SARS-CoV-2 are closely related to infection. Increasing fasting blood glucose levels after SARS-CoV-2 were reported by 30.7% (n = 55) of infected DM patients. Hyperglycemia is common in COVID-19 patients with and without DM due to direct injury of pancreatic β-cells caused by SARS-CoV-2 (61).

To our knowledge, this is the first study to investigate the actual COVID-19 vaccine coverage status and associated factors for vaccination behavior in DM patients in China. Our findings contributed to the endocrinologists’ understanding of the factors associated with COVID-19 vaccine behaviors in Chinese DM patients. There were still some limitations in this study. Firstly, our studies based on self-reported information might influence information validity. Although vaccination frequency and brand are based on vaccination information from the State Council APP, adverse reactions related to vaccination were all retrospective reports. Secondly, we did not investigate the information about blood glucose levels, medication, and other factors that could reflect the status of DM control. Further studies are needed to investigate the association between diabetes severity and COVID-19 vaccine uptake. Lastly, the causal conclusion could not be determined due to the cross-sectional study design.

## Conclusion

In the present study, the majority of DM patients (87.7%, n = 1929) have received at least one dose of the COVID-19 vaccine, while the coverage rate of full and booster vaccination (16.2%, n = 357; 65.2%, n = 1434) was relatively lower compared to the general population in China. Concerns about the safety of the COVID-19 vaccine were common in those unvaccinated and associated with the vaccine status. No severe adverse events occurred in this study, indicating that the COVID-19 vaccine was safe for DM patients.

## Data availability statement

The raw data supporting the conclusions of this article will be made available by the authors, without undue reservation.

## Ethics statement

The studies involving human participants were reviewed and approved by The Ethics Committee of Peking Union Medical College Hospital. The patients/participants provided their written informed consent to participate in this study.

## Author contributions

All authors contributed to the article and approved the submitted version. XX and YL conceived and designed the study. XX, ZW, JX, HJ, YC, JQ, HY, XZ, YL, XY, LX, XF, and SW contributed to data acquisition. HL and XL carried out the statistical analysis. All authors contributed to interpretation of data. HL wrote the first manuscript draft, which was critically revised by FP, YL and XX.
