# Supplementary material for: COVID-19 vaccine coverage, safety, and perceptions among patients with diabetes mellitus in China: a cross-sectional study
Source: Front Endocrinol (Lausanne). 2023 Jun 1;14:1172089. doi: 10.3389/fendo.2023.1172089 (PMC10270113; doi:10.3389/fendo.2023.1172089)
Supplement: Supplementary file 1 [file DataSheet_1.docx]

Table S1.The symptoms and characteristic of SARS-CoV-2 infected DM patients.

| Variables | SARS-CoV-2 infected participants  (n = 179) |
| --- | --- |
| **Method for determine infectivity (n, %)** |  |
| Reverse transcription PCR | 30 (16.8%) |
| SARS-CoV-2 antigen | 120 (67.0%) |
| Only through symptoms | 29 (16.2%) |
| **Duration of SARS-CoV 2 infection (day)** | 9 (7-10) |
| **Hospital visits (both inpatient- and outpatient-visits)** |  |
| Yes | 18 (10.1%) |
| No | 161 (89.9%) |
| **Symptoms (n, %)** |  |
| Fever | 151 (84.3%) |
| Fatigue | 104 (58.1%) |
| Headache | 59 (33.0%) |
| Muscle pain | 85 (47.5%) |
| Sore throat | 79 (44.1%) |
| Nasal congestion | 34 (19.0%) |
| Sniffles | 47 (26.3%) |
| Cough | 102 (57.0%) |
| Expectoration | 61 (34.1%) |
| Taste loss | 32 (17.9%) |
| Smell loss | 22 (12.3%) |
| Diarrhoea | 22 (12.3%) |
| Vomiting | 8 (4.5%) |
| Breathlessness | 21 (11.7%) |
| None | 4 (2.2%) |
| **Fasting blood glucose levels were increased (n, %)** |  |
| Yes | 55 (30.7%) |
| No | 59 (33.0%) |
| Unclear | 65 (36.3%) |
